# Investigating the incidence of drowning in different age groups in the universities of medical sciences in the first six months of 2020 and 2021

**Published:** 2023-12

**Authors:** Rita Motidost Komleh, Homa Yousefi Khoshsabegheh, Ali Davoudi Kiakalayeh

**Affiliations:** ^*a* Health Network Management Center, Ministry of Health, Tehran, Iran. ^; ^*b* Guilan Road Trauma Research Center, Trauma Institute, Guilan University of Medical Sciences, Rasht, Iran. ^

## Abstract

**Background:**

Although drowning is one of the 10 leading causes of death for people aged 1 to 24 in the world, it can be avoided. In Iran, children and teenagers are the most vulnerable victims of drowning.

**Methods:**

A cross-sectional descriptive approach was used. Drowning statistics, including both deaths and resuscitations, were collected from several sources in the first half of 2020 and 2021 including comprehensive health service centers, forensic medicine departments, network management center death registries, emergency services, hospitals, Red Crescent, and fire departments. The study analyzed various factors such as sex, place of drowning (including sea, river, pool, tub), and result of drowning (death and resuscitated) using statistical methods.

**Results:**

The number and rate of drowning increased from 977 people (1.16% of a thousand people) in 2020 to 1081 people (1.28% of a thousand people) in 2021. Moreover, 624 people (64%) and 685 people (63.37%) died in the first six months of 2020 and 2021, respectively. In the first half of 2020, 76.15% of the population were men and 23.85% women. In the same period of 2021, the percentage of men increased to 78.82% while that of women decreased to 21.18%. In Iran, June and July had the highest number of drownings in the first half of 2020 and 2021. The common places of drowning in 2020 and 2021 were the sea, river, swimming pool, bathroom, etc. It seems that drowning incidents had occurred in various places including barrels, water buckets, home ponds, construction ponds, wells, agricultural canals, sewage canals, aquariums, dams, sewage wells, and aqueducts.

**Conclusions:**

Drowning incidents in the first half of 2021 was more than that of the year 2020. Drowning prevention education, general education, recreational environment safety, and parent education are being recommended. The integration of a nationwide system for registering drowning incidents is necessary.

**Keywords:**

Injury, Drowning, Descriptive study

